# Immune Checkpoint Inhibitors Mediated Lymphocytic and Giant Cell Myocarditis: Uncovering Etiological Mechanisms

**DOI:** 10.3389/fcvm.2021.721333

**Published:** 2021-08-09

**Authors:** Rishi Rikhi, Jaret Karnuta, Muzna Hussain, Patrick Collier, Pauline Funchain, Wai Hong Wilson Tang, Timothy A. Chan, Rohit Moudgil

**Affiliations:** ^1^Department of Medicine, Cleveland Clinic Foundation, Cleveland, OH, United States; ^2^Department of Medicine, Case Western Reserve University, Cleveland, OH, United States; ^3^Department of Cardiovascular Medicine, Heart, Vascular and Thoracic Institute, Cleveland Clinic Foundation, Cleveland, OH, United States; ^4^Department of Hematology and Medical Oncology, Taussig Cancer Center Cleveland Clinic Foundation, Cleveland, OH, United States; ^5^Center for Immunotherapy and Precision Immuno-Oncology, Lerner Research Institute, Cleveland, OH, United States

**Keywords:** cardio-oncology, immune checkpoint inhibitor, immune-related adverse event, cardiovascular adverse event, myocarditis

## Abstract

The advent of immune checkpoint inhibitors (ICIs) has revolutionized the field of oncology, but these are associated with immune related adverse events. One such adverse event, is myocarditis, which has limited the continued immunosuppressive treatment options in patients afflicted by the disease. Pre-clinical and clinical data have found that specific ICI targets and precipitate distinct myocardial infiltrates, consistent with lymphocytic or giant cell myocarditis. Specifically, it has been reported that CTLA-4 inhibition preferentially results in giant cell myocarditis with a predominately CD4+ T cell infiltrate and PD-1 inhibition leads to lymphocytic myocarditis, with a predominately CD8+ T cell infiltrate. Our manuscript discusses the latest literature surrounding ICI pathways and targets, while detailing proposed mechanisms behind ICI mediated myocarditis.

## Introduction

Myocarditis is an illness caused by myocardial inflammation that can lead to heart failure (1). The etiology ranges from infectious, such as viral or bacterial, to non-infectious, including autoimmune and drug toxicity (1). Similarly, the clinical presentation is heterogeneous and can mimic other pathologies, such as acute coronary syndrome (1–3). However, a diagnosis of fulminant myocarditis portends a grave prognosis with a high rate of fatality and serious morbidity due to electrical instability and acute decompensated systolic heart failure, requiring circulatory support for mechanical compromise (2).

Current treatment guidelines to manage myocarditis are similar to management of chronic systolic heart failure, as prior immunosuppressive strategies have failed to show any significant difference (2, 4). Heart failure treatments for myocarditis are aimed at preserving left ventricular function and improving left ventricular remodeling (5). Chronic inflammation from myocarditis can lead to myocyte death, fibrosis, and scarring, ultimately leading to a dilated cardiomyopathy (5). In order to provide more effective treatments, mechanisms involved in the development of myocarditis need to be understood. At the present time, targeted therapies for myocarditis are lacking, in part due to a gap in understanding of the pathophysiology and precipitating factors involved in the development of myocarditis (1). A recently published consensus highlights these deficiencies and while it provides a template for potential therapies, strong mechanistic data is still lacking (5).

The recent advent of immune checkpoint inhibitors (ICIs) has highlighted a possibility of elucidating mechanisms behind the development of these individual myocarditis subtypes. ICI therapies have revolutionized cancer treatment and improved survival in many oncological diseases such as melanoma of the skin, non-small cell lung cancer, kidney cancer, bladder cancer, head and neck cancers, and Hodgkin lymphoma (6). ICIs act on T cells leading to immune system activation and subsequent attenuation, if not amelioration, of cancer (7, 8). However, 60–80% of patients experience some degree of autoimmune inflammatory responses due to a heightened immune response, grouped under immune related adverse events, that can affect almost any organ system (7, 9). Cardiovascular adverse events (CVAEs) due to ICIs include arrhythmias, cardiomyopathies, coronary artery disease, pericarditis, myocarditis and as we identified recently, valvulitis (7, 10). Of the CVAEs, the most common, approaching 45% of all CVAEs, and the condition that poses the greatest threat on life, is myocarditis (7). Current data estimate the prevalence of ICI-mediated myocarditis to range from 0.06 to 2.4% (11). Additionally, recent studies have shown that myocarditis has been presenting with other pathologies such as myositis and myasthenia gravis, termed 3M (12, 13). The mortality rate in the last cohort is estimated at 52% and is largely driven by humoral response to ICI (14). Recent literature has shown that ICI therapy leads to myocarditis, specifically lymphocytic myocarditis (LM) and giant cell myocarditis (GCM), with distinct immune profiles (15–17). The objective of this review is to shed light on the mechanism underlying the development of ICI-mediated LM and GCM.

## Current Paradigm of Potential Mechanisms

ICI-mediated myocarditis may present in the form of LM as well as GCM, and in order to better understand their potential mechanisms, it is important to review the current knowledge about mechanisms driving these pathologic findings.

### Lymphocytic Myocarditis

LM is typically characterized by patchy infiltration of an array of mononuclear cells, with a majority of T lymphocytes and some macrophages (1, 18). Other immune related cells, such as neutrophils, eosinophils, and plasma cells may be present, but are not the prominent cell type (1). LM is further characterized into acute and chronic LM based on immunopathology (18). In acute LM, there is minimal fibrosis, however chronic LM is characterized by fibrosis given the persistent inflammation (18). Of note, in chronic LM, the lymphocytic infiltration still remains in addition to fibrosis (18). Currently, the timing of transition from acute to chronic LM is not well described and varies at the individual level (18). Thus far, studies have found LM is due to viral (coxsackie B virus and adenovirus being the most common) and autoimmune etiologies (1, 18). The exact pathophysiology of LM is not completely understood; however, current proposed mechanisms have been attributed to an inflammatory response following viral infection (19).

It has been identified that viral infection causes activation of toll-like receptors which leads to an innate immune response and upregulation of immune mediators (19). As the inflammatory cascade continues, the acquired immune system is eventually generated, leading to T cell activation (19). In order for T cell activation to occur, viral antigen must be presented via binding between the T cell receptor and major histocompatibility complex (19, 20). If the viral antigen peptide shares similarity to myosin, or other myocardial antigens, the T cells may attack the myocardium, which is known as molecular mimicry (19, 20). Through damage from T cells, the myocardium exposes more antigens and provokes further activation of toll-like receptors, perpetuating the immune response (19). This continued inflammatory response engenders B cell activation and production of cardiac autoantibodies, leading to further myocardial inflammation and damage (19). Studies have found that this pathologic immune response is regulated by T helper 17 (Th17) cells and regulatory T cells (19). Th17 cells lead to a proinflammatory state while regulatory T cells lead to an immunosuppressive state and are thus responsible for increasing the threshold of immune cells in recognizing self-antigens (19). Demonstrating this effect, research has shown that humans diagnosed with myocarditis have higher ratios of Th17 to regulatory T cells in peripheral blood, leading to proinflammatory cytokines and the recruitment of inflammatory cells to the myocardium (19). Also, in animal models, injection with regulatory T cells has been shown to decrease inflammation in viral myocarditis (19). Despite the implications of various pathways, a coherent immune-pathological mechanistic understanding of LM is lacking.

### Giant Cell Myocarditis

GCM is another histological subtype with a more severe clinical course and worse prognosis (1). The average age at diagnosis is 40 and approximately 20% of patients have an autoimmune condition (19). Histology of GCM is comprised of myocyte necrosis with T lymphocytes and multinucleated giant cell derived from macrophages (1, 21). Eosinophils and plasma cells may be found, but to a much smaller degree (1). The pathophysiology of GCM is not fully understood, but current evidence suggests an autoimmune etiology (22). The initial support of an autoimmune etiology for GCM came from basic science research where mice were injected with either membranous proteins or cardiac myosin (22). In this study, only the mice injected with cardiac myosin developed myocarditis with histology showing myocardial necrosis and giant cells (22). Thus, cardiac myosin was able to elicit an autoimmune-mediated myocarditis, with histology consistent with GCM (22). Further insight into the pathogenesis of GCM was offered with gene expression analysis, which has helped to characterize the specific immune response involved in GCM (23). In a study comparing gene expression of GCM vs. normal heart tissue samples, the authors found a large number of genes T cell activation genes upregulated in GCM samples (23). More specifically T helper type 1 (Th1) cells, were involved in precipitating the immune response in GCM (23). The predominantly T cell response in GCM is different from LM, where autoimmune damage in the latter occurred in part through B cells and cardiac autoantibodies (19).

## ICI Signaling Pathways

**Figure 1:**
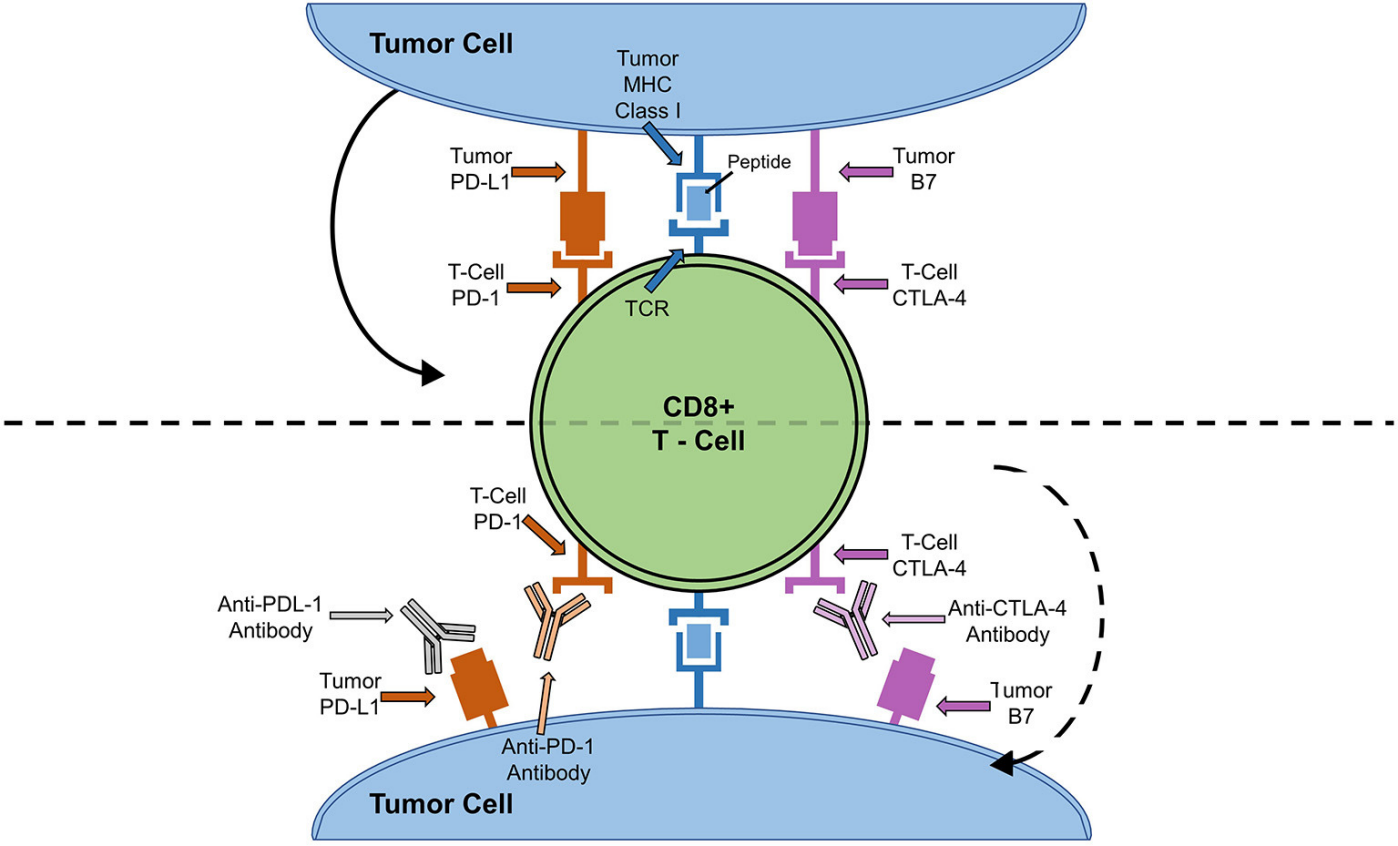
ICI Cell Signaling Pathway and Targets. Solid arrow depicts how binding of tumor cell PD-L1 to PD-1 or B7 to CTLA-4 leads to down-regulation of T-cell activity. Dotted arrow depicts how blocking of T-cell anergy receptor ligands (B7 and PD-L1) results in T cell activation and tumor killing.

ICIs were developed under the premise that cancer cells escape the immune system by exploiting negative feedback mechanisms on the surface of T cells (6). ICI prevents engagement of these pathways and harnesses the power of the immune system to kill cancer cells (6). In order to understand the mechanisms by which ICIs work, it is important to understand the cytotoxic T-lymphocyte antigen 4 (CTLA-4) and programmed cell death protein (PD-1) - program death cell protein ligand (PD-L1) signaling pathways (Figure 1) (24).

T cell activation is a complex and dynamic process of inhibitory and stimulatory signaling (7, 24). In order for T cells to become activated, co-receptors on T cells must interact with major histocompatibility complexes located on antigen presenting cells (7, 24). CTLA-4 and PD-1 on T cells bind to B7 and PD-L1 on antigen presenting cells, respectively (7, 24). This interaction results in an inhibitory signal, preventing T cell activation and proliferation (7, 24). Thus, by blocking CTLA-4, PD-1, or PD-L1, T cells are activated to ward off cancer cells (7, 24). Until recently, there have been seven FDA approved ICIs: ipilimumab, monoclonal antibody against CTLA-4, pembrolizumab, nivolumab and cemiplimab, monoclonal antibodies against PD-1, and atezolizumab, durvalumab, and avelumab, monoclonal antibodies against PD-L1 (Table 1) (25, 28). Earlier this year, the FDA granted breakthrough therapy designation of tiragolumab, an anti-T cell immunoreceptor with Ig and immunoreceptor tyrosine-based inhibitory motif domains (TIGIT), for use in PD-L1-high non-small cell lung cancer (Table 1) (31, 32). A thorough review of the ICI mediated mechanisms can be found by Wei et al. (33).

**Table 1:** Immune checkpoint inhibitors and molecular targets.

**Immune checkpoint inhibitor**	**Molecular target**	**Associated myocarditis immune profile**
Ipilimumab (16, 25)	CTLA-4	GCM, CD4+ T cell infiltration
Pembrolizumab (25, 26)	PD-1	LM, CD8+ T cell infiltration
Nivolumab (25, 27)	PD-1	LM, CD8+ T cell infiltration
Cemiplimab (28)	PD-1	LM, CD8+ T cell infiltration
Atezolizumab (25, 29)	PD-L1	LM, CD8+ T cell infiltration
Durvalumab (25, 30)	PD-L1	LM, CD8+ T cell infiltration
Avelumab (25)	PD-L1	LM, CD8+ T cell infiltration
Tiragolumab (31, 32)	TIGIT	Not documented

### ICI-Mediated Myocarditis Mechanisms

To date, the exact mechanism of the development of myocarditis from ICI therapy is not understood (24, 34). However, novel research has focused on better elucidating the mechanisms of ICI-mediated myocarditis (17). Wei et al. provided insight on distinct cellular mechanisms of anti-CTLA-4 and anti-PD-1 on human melanoma and murine tumor models (17). The authors showed that anti-PD-1 therapies resulted in expansion of exhausted-like tumor infiltrating CD8+ T cells (17). This is in line with other studies which have also shown that dynamic expansion of CD8+ T cells takes place with PD-1 inhibition (35, 36). Conversely, anti-CTLA-4 therapies result in expansion of Inducible T cell CO-Stimulator Th1-like CD4 effector as well as exhausted-like CD8+ T cells (17). The expansion of Inducible T cell CO-Stimulator CD4+ T cells has been seen with CTLA-4 inhibition in various tumor subtypes, also confirming the preclinical findings of Wei et al. (17, 37–39). These results illustrate how anti-CTLA-4 and anti-PD-1 therapies results in unique cellular mechanisms (17).

Preclinical research has shown that CTLA-4 and PD-1 play a key role in peripheral immune tolerance, and disruption leads to activation T lymphocytes targeting cardiac antigens (24). Murine studies have shown that antibodies against CTLA-4 or CTLA-4 deficient murine models both result in the clinical picture of myocarditis (40–42). Similarly, murine models deficient in PD-1 and PD-L1 lead to myocarditis and dilated cardiomyopathy as well (43–45). In fact, one study investigating PD-1 knockout mice found myocarditis in 96% of the murine models (18). Another study delivered cardiac troponin antibodies to murine models deficient in PD-1, leading to a dilated cardiomyopathy, further highlighting the immunoregulatory significance of PD-1 and PD-L1 (46). This is of grave concern as T cells that escape negative selection, and may have reactivity to myocardial tissue, are now at a lower threshold for activation, leading to myocardial infiltration (18, 24).

In addition to murine models, there have been clinical studies examining immune profiles of patients with ICI-mediated myocarditis. Reuben et al. described a case of a patient with metastatic melanoma treated with ipilimumab who developed steroid refractory GCM (16). Immune analysis of post-mortem cardiac tissue by immunohistochemistry, T cell receptor sequencing, and gene expression profiling were conducted (16). Autopsy demonstrated a myocardial infiltrate composed of lymphocytes, multinucleated giant cells, and some eosinophils (16). The myocardium was cultured and negative for any infectious etiology. Immunohistochemical staining revealed predominantly CD4+ T cells in the heart (16). Forkhead box protein P3 (FoxP3) staining was used to identify regulatory T cells and CD45RO was used to identify antigen-experienced T cells (16). FoxP3 and CD45RO quantification showed few FoxP3 regulatory T cells in the heart, liver and lung metastasis with CD45RO expression predominant in the heart (16). These results demonstrate that CD4+ T cells were predominant in GCM with a concomitant increase expression of CD45RO+ cells (16). Thus, ipilimumab resulted in CD4+ T cell infiltration into the myocardium, resulting in the development of GCM (16).

**Figure 2:**
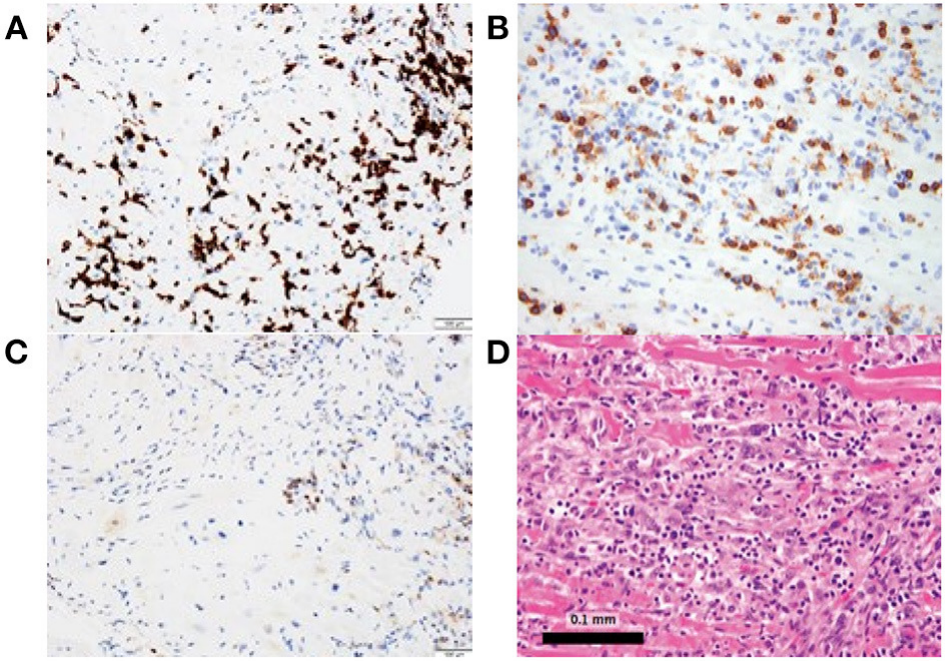
Myocardial biopsy depicting lymphocytic myocarditis in a patient who received both nivolumab and ipilimumab. **(A, B)** Immunostaining is predominantly CD8+, **(C)** with CD4+ staining and **(D)** hematoxylin and eosin staining.

Conversely, Johnson et al. described two cases of patients with melanoma treated with combination of ipilimumab and nivolumab who developed fatal LM (15). Both patients had immune infiltration affecting only the cardiac and skeletal muscle cells (15). Postmortem evaluation of these two patients revealed myocardium infiltrates with both CD4+ and CD8+ T cells (Figure 2) (15). Similarly, Laubli et al. discussed a case of patient with melanoma who was treated with pembrolizumab resulting in myocarditis (26). Infectious workup was negative and myocardial biopsy showed lymphocytic infiltration with predominantly CD8+ T cells (26). Thus, PD-1 inhibitor alone or in combination with CTLA-4 inhibitor mediates LM primarily comprised of CD8+ T cells; with immune profiling distinct from GCM patients (15, 26).

The above preclinical and clinical studies highlight that CTLA-4 inhibition may cause development of GCM with predominantly CD4 infiltration while PD-1 inhibition preferentially results in the development of LM with predominantly CD8 infiltration. Hence, different ICI combinations precipitate distinct forms of myocarditis with a unique pattern of immune infiltration, a mechanism which may not necessarily translate into non-ICI myocarditis. This differential T cell infiltrative response to CTLA-4 inhibition PD-1 inhibition could be in part due to cytokine activation (47). Chemokines help with recruitment and T cell trafficking, and thus dictate the immune profiles of inflammatory processes (47). C-X-C Motif Chemokine Receptor 3 (CXCR3) is involved in several pathways, including mitogen-activated protein (MAP) kinases and phosphoinositide 3-kinase (PI3K)/ protein kinase B (Akt), leading to activation, differentiation, and recruitment of CD4+ T cells (48, 49). CXCR3 appears to favor recruitment of CD4+ T cells compared to CD8+ T cells (50). Additionally, increased expression of CXCR3 and its chemokine ligands have been found in GCM from CTLA-4 inhibition (16). Conversely, C-C chemokine receptor type 5 (CCR5) has been shown to recruit CD8+ T cells in response to chemokine ligand 3 (CCL3) and chemokine ligand 4 (CCL4), a mechanism that has also been found in other cardiovascular conditions (51, 52).

Similar recruitment has been demonstrated in Chagas disease, which is caused by *Trypanosoma cruzi* (52). Histologically, the non-ischemic cardiomyopathy in Chagas disease is characterized by a predominantly CD8+ T cell infiltrate (19, 52). In a murine study of Chagas disease, CCR5 and its chemokine ligands aided in T cell migration to cardiac tissue (52). Another example highlighting the importance of CCR5 in CD8+ T cell recruitment is Kawasaki disease, which is a fatal vasculitis in children that can lead to coronary artery aneurysms (53, 54). Although the exact mechanism of Kawasaki disease is not clear, histology is consistent with a predominantly CD8+ T cell infiltrate (54). Further, genetic haplotypes that result in loss of CCR5 expression have been shown to be inversely related to Kawasaki disease incidence (53). These findings further illustrate the role of CCR5 in CD8+ T cell recruitment (53). Thus, CD4+ versus CD8+ T cell infiltrate could be partially explained by differential chemokine response, specifically CXCR3 versus CCR5 in development of GCM or LM, respectively.

In addition to chemokine recruitment, studies have found the immunoproteasome to lead to CD4+ T cell recruitment (55). In a study by Bockstahler et al., murine models of autoimmune myocarditis were treated with an immunoproteasome inhibitor (55). Here, the immunoproteasome was found to increase cytokine production, which led to a decrease in T regulatory cells and differentiation of CD4+ T cells to Th1 and Th17 cells (55). The authors found that blocking the immunoproteasome led to a significant reduction in the autoimmune response and subsequent myocarditis (55). Thus, given the predominantly CD4+ T cell response, the immunoproteasome may play a key role in the development of GCM and myocarditis caused by ICIs directed at CTLA-4.

While the above pre-clinical and clinical data demonstrate the complex interplay between ICI, chemokine signaling, and distinct immune myocardial infiltrates, the molecular mechanism behind injury to cardiomyocytes is thought to be due to a shared antigen (34). As mentioned above, Johnson et al. discussed two cases of melanoma treated with ICI who developed fatal myocarditis (15). Interestingly, the affected tissues were cardiac muscle, skeletal muscle, and tumor, all with T cell infiltrates that had similar T cell receptor homology (15). Additionally, histology of the tumor samples from the two patients demonstrated desmin and troponin, which are antigens found in muscle, further illustrating the possibility of a shared antigen leading to ICI mediated myocarditis (15, 34). Recent case reports have shown that anti-striated muscle antibody might be a key factor as the elevation of this antibody and subsequent decrease with intravenous immunoglobulin (IVIG) can result in improvement in clinical outcomes (12).

### ICI Mediated Myocarditis Clinical Characteristics

With the expanding use of ICIs, data on the clinical features of ICI mediated myocarditis continues to expand (56). Mahmood et al. conducted a retrospective case-control study comparing 35 cases of myocarditis due to various ICIs to 105 control patients who received ICIs, but did not develop myocarditis, from 2013 to 2017 (56). Data from this study showed that the average time from initiation of ICI to myocarditis presentation was 34 days, 94% of cases had troponin elevation, 89% of cases had an abnormal electrocardiogram, 51% of cases had a normal ejection fraction, and 46% of cases had major adverse cardiac events (56). Interestingly, of the 46% of cases that developed major adverse cardiac events, 38% had a normal left ventricular ejection fraction (56). Compared to non-ICI mediated myocarditis cases, data from this study suggest that a higher percentage ICI mediated myocarditis cases develop a more severe course, and of those that develop a fulminant course, a higher percentage have a normal ejection fraction (56–58).

Also of growing interest is the association of specific ICI therapies to myocarditis prevalence and outcomes. Studies have shown that combination therapies lead to higher rates of myocarditis, as well as worse outcomes (59). For example, one study showed that nivolumab (PD-1 inhibitor) and ipilimumab (CTLA-4 inhibitor) had almost a 5 times greater incidence of myocarditis compared to single agent nivolumab (59). In another study, Moslehi et al. examined 101 cases of ICI mediated myocarditis, and mortality rates were significantly higher among individuals who received combination ICI therapy versus monotherapy (67 vs. 36%, respectively) (60). Further, Chen et al. conducted a retrospective study using FDA Adverse Event Reporting System and found a stronger association of PD-1 inhibitors and myocarditis compared to CTLA-4 and PD-L1 inhibitors (61). As more data becomes available, we will have a better understanding on the prevalence and outcomes of myocarditis with specific ICI therapy.

## Implications of Therapy

Current recommendations for ICI mediated myocarditis treatment are based on expert opinion and case series given that clinical trials and prospective studies have not occurred at this time (62). Once ICI mediated myocarditis is suspected, the first step is to hold further ICI therapy, followed by administering corticosteroids (62). The optimal dose of steroids has not been established; however, data currently shows that higher doses of steroids lead to less major adverse cardiac events (56). Current recommendations suggest starting treatment with prednisone 1–2 mg/kg and treatment with 500–1000 mg of methylprednisolone for severe or refractory cases (59, 63). If there is no response to steroid therapy, infliximab or mycophenolate mofetil are further immunosuppressive medications that can be used (63). More recently, translational research on the use of abatacept by Salem et al. has been reported (64, 65). Abatacept is an agonist toward CTLA-4, preventing costimulation of T cells, and was used in a case of steroid refractory nivolumab induced myocarditis, resulting in a lifesaving treatment (64, 65). There is a strong need for prospective treatment studies and immunosuppressive strategies that target the underlying mechanism behind the development of ICI mediated myocarditis.

Based on the current understanding of the pathogenesis and immune profiles of both LM and GCM, potential mitigating strategies exist (Table 2). Perhaps the most important study in the consortium of trials was the Myocarditis Treatment Trial conducted in 1995 (4). After evaluation, 111 patients were enrolled in this trial with a histopathological diagnosis of myocarditis and a left ventricular ejection fraction of <0.45 to either receive conventional therapy alone or combined with a 24-week regimen of immunosuppressive therapy (4). Immunosuppressive therapy consisted of prednisone with either cyclosporine or azathioprine (4). The primary outcome measure was a change in the left ventricular ejection fraction at 28 weeks (4). After 28 weeks, there were no changes in the primary outcome (4). Additionally, no change in survival rates were noted (4). Unfortunately, like the Myocarditis Treatment Trial, other trials that have looked at immunosuppressive therapies lacked the knowledge of underlying etiologies of the enrolled myocarditis patients (69–72). Therefore, a comprehensive immunosuppressive guideline as it pertains to myocarditis remains to be determined.

**Table 2:** Select studies highlighting use of various immunosuppression strategies in myocarditis.

**Study**	**Patients**	**Criteria**	**Immunosuppression**	**Outcomes**	**Conclusion**
The Myocarditis Treatment Trial (4)	111	Histopathological diagnosis and Left Ventricular Ejection Fraction (LVEF) <0.45	Prednisone with cyclosporine or azathioprine	Change in LVEF 28 weeks	No statistical difference in EF or survival
GCM Treatment Trial (66)	12	Biopsy-proven GCM with <3 months of symptoms	Steroids and cyclosporine +/– muromonab-CD3	EMB and LVEF in 4 weeks	Statistically significant decrease in inflammatory cells and no statistical difference in EF
Rabbit anti-thymocyte globulin (RATG) at Harefield Hospital (67)	6	Histopathological diagnosis	RATG + methylprednisolone	Mean LVEF improvement	Mean LVEF improvement 29%
Intervention in Myocarditis and Acute Cardiomyopathy (IMAC) trial (68)	62	Histopathological diagnosis (cellular inflammation not necessary, GCM excluded), LVEF <0.4, <6 months of symptoms without CAD or valvular disease	IVIG	LVEF change at 6 and 12 months	No statistical difference in change in EF compared to placebo

In a prospective trial involving 12 subjects with GCM, immunosuppression led to decreased cellular myocardial inflammation (66). The study used prednisone and cyclosporine, with some subjects receiving muromonab-CD3 in addition (66). Muromonab-CD3 is a monoclonal antibody that targets CD3, preventing T cell proliferation (66). Thus, Muromonab-CD3 has the potential to dampen the predominantly a CD4+ T cell response in GCM (22, 66). Since its initial approval in 1986, Muromonab has been discontinued given other therapeutic options with better side effect profiles (73). Another method of suppressing the T cell response is with anti-thymocyte globulin, which consists of rabbit or horse derived polyclonal IgG antibodies against human T cells (67, 74). In a retrospective study of 6 patients with GCM who were treated rabbit anti-thymocyte globulin in conjunction with steroids, there was an average improvement in left ventricular ejection fraction of 29% (67). Additionally, five of the six patients in the study were discharged home from the hospital and at follow up of about 1,100 days, three patients were alive without recurrence and had an average left ventricular ejection fraction of 55% (67). The above therapies are the first to provide a mechanistic window of potential strategies for attenuating and ameliorating GCM, which is of incredible value as the average survival without transplant or immunosuppression in GCM is 12 weeks (75). Similar therapeutic strategies can be applied to LM in order to mitigate the CD8+ T cell response. Success has been seen in Kawasaki disease, mentioned above, where IVIG is used in acute treatment and has been found to lower the incidence of coronary artery aneurysms by 20% (76). The mechanism behind IVIG is thought to be upregulation of Interleukin-10 (IL-10) and regulatory T cells, suppressing the immune response (76). Thus, IVIG is used to dampen the CD8+ T cell inflammatory response in Kawasaki disease and has the potential to mitigate the CD8+ T cell inflammatory response seen in LM (76). However, the Intervention in Myocarditis and Acute Cardiomyopathy (IMAC) trial, a prospective placebo-controlled trial, investigated 62 patients with less than a 6-month onset of dilated cardiomyopathy and found no additional improvement in ejection fraction with IVIG compared to placebo (68). While results were equivocal, further research could investigate alternate, and possibly, more prolonged dosing schedules of IVIG (68).

## Conclusions

Although several causes of myocarditis have been established, etiology-specific treatment strategies are limited (1, 2). Additionally, a critical void exists in understanding the mechanistic pathway involved in the development of myocarditis. The innovation of ICI has provided insight to understanding the immune profiles involved in myocarditis. Specifically, studies have found CTLA-4 inhibition causes GCM with a predominately CD4+ T cell infiltrate. Conversely, PD-1 inhibition leads to LM, with a predominately CD8+ T cell infiltrate. These distinct immune profiles from CTLA-4 and PD-1 inhibition offer opportunities to characterize the mechanism behind ICI-mediated myocarditis. Further research is needed to explore T cell repertoire and chemokine patterns involved that precipitate different forms of myocarditis. These studies could result in promising new avenues of therapeutic targets in the field of ICI and non-ICI-mediated myocarditis.

## Author Contributions

WT, TC, and RM: concept and final approval. RR, JK, and MH: draft of manuscript. PC, PF, WT, and TC: critical revision of article. RR, JK, MH, PC, PF, WT, TC, and RM: literature review. JK: figures. RR: tables. All authors contributed to the article and approved the submitted version.

## Conflict of Interest

The authors declare that the research was conducted in the absence of any commercial or financial relationships that could be construed as a potential conflict of interest.

## Publisher's Note

All claims expressed in this article are solely those of the authors and do not necessarily represent those of their affiliated organizations, or those of the publisher, the editors and the reviewers. Any product that may be evaluated in this article, or claim that may be made by its manufacturer, is not guaranteed or endorsed by the publisher.
